# Experiments and Observations on Cinchona, Tending Particularly to Shew That It Does Not Contain Gelatine

**Published:** 1804-03-01

**Authors:** Andrew Duncan


### Experiments *and* Observations *on* Cinchona, *tending particularly to shew that it does not contain* Gelatine, *by* Andrew Duncan, M.D. F.R.S.E.

Having been long engaged in a series of experiments on the astringent substances employed in medicine, I was particularly interested with "a Memoir on the Febrifuge Principle of Cinchona," lately published. The presence of gelatine in cinchona, was so incompatible with experiments I had

I had formerly made, that I was strongly inclined to believe, that Seguin (than whom no one should be better acquainted with the combinations of tannin and gelatine) had been misled, either from having examined cinchona which had been adulterated, or from some other accidental cause. To satisfy myself I immediately proceeded to the unerring test of experiment, which has convinced me that cinchona does *not* contain gelatine, but some other principle not yet sufficiently examined, which agrees with gelatine, in forming with tannin, a precipitate comparatively insoluble in water. At the same time it is but fair to remark, that my experiments were made with the infusion and tincture of cinchona, containing all the soluble principles of that substance, whereas Seguin's observations are said to be derived from the examination of the isolated febrifuge principle, of which he gives the following characters: "It precipitates the solution of tan, but not the solutions of gelatine and sulphate of iron." On the contrary, my experiments teach me, that the entire infusion and tincture of cinchona, precipitate the solution of tan, and also the solution of gelatine slightly, and the solution of sulphate of iron copiously. But as the two last precipitates may be reasonably ascribed to the action of other principles contained in my infusion and tincture of cinchona, I shall not insist upon them, but proceed to shew that, although cinchona actually does precipitate the solution of tan, yet it does not contain gelatine.

Exp. I. 1. An ounce of infusion of galls was saturated, by adding to it in different portions, an ounce and a half of infusion of cinchona. The mixture was white and turbid, with a loose light precipitate.

2. On filtration the fluid passed almost colourless, and perfectly transparent.

3. The precipitate when dried, weighed five grains. It had a yellow colour, and an opake earthy appearance, was extremely friable, and did not adhere to the filtering paper.

4. The filtred fluid gave no further precipitate with solution of cinchona, but with half an ounce of solution of gelatine, containing six grains of gelatine in each ounce, it produced a copious precipitate, and was saturated.

5. The precipitate, when separated by filtration, and dried also, weighed five grains, but was hard and brittle, adhered strongly to the paper, had a yellow colour, and exactly resembled a resin in appearance.

Exp. II.

Exp. II. 1. An ounce of the same infusion of galls was saturated by an ounce and a half of the same solution of gelatine. Immediately a very copious, whitish, tenacious, and adhesive precipitate was formed.

2. On filtration the fluid passed very slowly; and even after repeated filtration, still retained a slight degree of opaline bluishness.

3. The precipitate when dried, weighed fourteen grains and a half. It had a brownish yellow colour, was transparent, and had a resinous appearance and fracture. It was also hard and brittle, and adhered strongly to the filter. In every particular it resembled the precipitate produced in the former experiment (Exp. I. 1) by gelatine, after the infusion of galls was completely saturated by cinchona.

4. In the filtred liquor (Exp. II. 2) infusion of cinchona produced no change.

Exp. III. To an ounce and a half of the same infusion of cinchona, half an ounce of the solution of gelatine was added. It produced only a slight degree of turbidness, and changed the colour of the infusion from a pale greenish to a reddish yellow colour. When filtred, it passed perfectly transparent, and the bottom of the filter was covered with a red varnish; but it had gained only one grain in weight. In other experiments with larger quantities and stronger infusion of cinchona, the presence of tannin was more strongly indicated.

Exp. IV. Infusion of galls was not affected by rectified spirits of wine, in which isinglass had been long infused.

Exp. V. 1. A tincture of cinchona was prepared by infusing it in the same rectified spirits. After it was filtred some resin was separated by precipitation with water and filtration.

2. With infusion of galls this tincture gave a copious precipitate, exactly resembling that produced by the same reagent and infusion of cinchona. (Exp. I. 3.)

Exp. VI. With tincture of galls the same tincture of cinchona gave no precipitate.

Exp. VII. In the mixed tincture (Exp. VI.) a copious precipitate was produced by diluting it with water.

Exp. VIII. A solution of carbonate of potash produced a copious white flaky precipitate in the solution of gelatine, which was soluble in boiling water, but was not precipitated from the solution by infusion of galls, until some acid was added.

Exp. IX.

Exp. IX. The solution of carbonate of potash changed the colour of the infusion of cinchona to a fine red, without disturbing its transparency.

These facts seem to me sufficient to prove the difference between gelatine, and the new principle in cinchona, which for the sake of convenience, I shall venture for the present to denominate cinchonin.

Gelatine is soluble in water, and the solution is disposed to gelatinize. Six grains of isinglass, dissolved in one ounce of water, form with it at temperatures below 60° Fahrenheit, a jelly of considerable firmness. From its solution in water, gelatine is precipitated by alcohol, and a solution of carbonate of potash. It is precipitated also by tannin, and the precipitates form a hard brown transparent mass.

Cinchonin is soluble in water, but gives it no tendency to gelatinize. From its solution in water, it is not precipitated by a solution of carbonate of potash. It is soluble in alcohol; it combines with tannin. The compound is soluble in alcohol, but forms, when water is added, or used as a menstruum, a friable opaque yellowish precipitate; but cinchonin does not separate even from a watery solution of tanin, all that is precipitable by a solution of gelatine.

